# A second polymorph of [1,2-bis­(di-*tert*-butyl­phosphino)ethane]dichlorido­platinum(II)

**DOI:** 10.1107/S1600536808000603

**Published:** 2008-02-06

**Authors:** Ahmet Gunay, William W. Brennessel, William D. Jones

**Affiliations:** a Department of Chemistry, University of Rochester, Box 270216, Rochester, NY 14627-0216, USA

## Abstract

The title complex, [PtCl_2_(C_18_H_40_P_2_)], contains a Pt^II^ center in an approximately square-planar geometry [*cis *angle range = 88.09 (3)–91.39 (3)°; twist angle = 1.19 (5)°]. The Pt—P bond lengths of 2.2536 (8) and 2.2513 (8) Å and the Pt—Cl bond lengths of 2.3750 (8) and 2.3588 (8) Å are normal. This crystal form is a polymorph of a structure reported previously [Harada, Kai, Yasuoka & Kasai (1976 ▶). *Bull. Chem. Soc. Jpn*, **49**, 3472–3477].

## Related literature

For related literature, see: Crascall & Spencer (1990 ▶); Green *et al.* (1977 ▶); McDermott *et al.* (1976 ▶); Ogoshi *et al.* (2004 ▶).
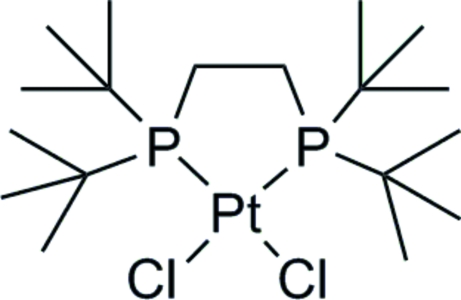


## Experimental

### 

#### Crystal data

[PtCl_2_(C_18_H_40_P_2_)]
*M* *_r_* = 584.43
Monoclinic, 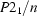

*a* = 11.0981 (10) Å
*b* = 15.3242 (13) Å
*c* = 14.5413 (13) Å
β = 109.287 (1)°
*V* = 2334.2 (4) Å^3^
*Z* = 4
Mo *K*α radiation
μ = 6.38 mm^−1^
*T* = 100.0 (1) K
0.20 × 0.14 × 0.08 mm

#### Data collection

Bruker SMART APEXII CCD diffractometer
Absorption correction: multi-scan (*SADABS*; Sheldrick, 2007 ▶) *T*_min_ = 0.342, *T*_max_ = 0.600
20415 measured reflections
8022 independent reflections
6312 reflections with *I* > 2σ(*I*)
*R*_int_ = 0.034

#### Refinement

*R*[*F* ^2^ > 2σ(*F* ^2^)] = 0.029
*wR*(*F* ^2^) = 0.060
*S* = 1.01
8022 reflections
208 parameters
H-atom parameters constrained
Δρ_max_ = 1.11 e Å^−3^
Δρ_min_ = −0.81 e Å^−3^

### 

Data collection: *APEX2* (Bruker, 2006 ▶); cell refinement: *SAINT* (Bruker, 2006 ▶); data reduction: *SAINT*; program(s) used to solve structure: *SIR97* (Altomare *et al.*, 1999 ▶); program(s) used to refine structure: *SHELXL97* (Sheldrick, 2008 ▶); molecular graphics: *SHELXTL* (Bruker, 2000 ▶); software used to prepare material for publication: *SHELXTL*.

## Supplementary Material

Crystal structure: contains datablocks I, global. DOI: 10.1107/S1600536808000603/pv2062sup1.cif

Structure factors: contains datablocks I. DOI: 10.1107/S1600536808000603/pv2062Isup2.hkl

Additional supplementary materials:  crystallographic information; 3D view; checkCIF report

## Figures and Tables

**Table:** 

Pt1—P2	2.2513 (8)
Pt1—P1	2.2536 (8)
Pt1—Cl2	2.3588 (8)
Pt1—Cl1	2.3750 (8)

**Table:** 

P2—Pt1—P1	89.70 (3)
P2—Pt1—Cl2	90.82 (3)
P1—Pt1—Cl2	178.77 (3)
P2—Pt1—Cl1	178.84 (3)
P1—Pt1—Cl1	91.39 (3)
Cl2—Pt1—Cl1	88.09 (3)

**Fig. 1.**
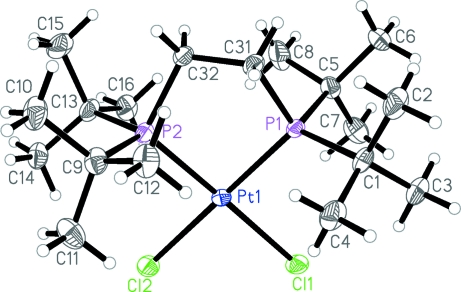
Displacement ellipsoid (50% probability) drawing of (I) with H atoms omitted.

## supplementary crystallographic information

### Comment

One of the most commonly used Pt(0) precursors, Pt(COD)_2_, COD = 1,5-cyclooctadiene, is generally synthesized by the reduction of platinumdichlorides, like Pt(COD)Cl_2_, with Li_2_(COT), COT = cyclooctatetraene (Green *et al.*, 1977; Crascall & Spencer, 1990), or with SmI_2_ (Ogoshi *et al.*, 2004). The latter reduction with 20 equivalents of SmI_2_ afforded Pt(COD)_2_ in moderate yields (45% average). After addition of chelating ligand 1,2-bis(di-*tert*-butylphosphino)ethane (dtbpe) to the SmI_2_ reduction product, it was observed that some Pt^II^ remained, based on the formation of the title compound, Pt(dtbpe)Cl_2_ (I). An independent synthesis of (I) was performed to support these observations, in which dtbpe was added directly to Pt(COD)Cl_2_ (see experimental section). The resulting pure product in 88% yield was characterized by ^1^H, ^13^C, ^31^P NMR spectroscopies and by single-crystal X-ray diffraction.

### Experimental

Pt(COD)Cl_2_, COD = 1,5-cyclooctadiene, was synthesized according to the published procedure (McDermott *et al.*, 1976). Under an atmosphere of dinitrogen, bis(di-*tert*-butylphosphino)ethane (dtbpe) (212 mg, 0.67 mmol) was added to a light yellow suspension of Pt(COD)Cl_2_ (250 mg, 0.67 mmol) in THF (25 ml). The reaction mixture was heated with stirring for 12 h at 373 K. After complete conversion to (I) was verified by ^31^P NMR spectroscopy, the volatiles (THF, COD) were removed *in vacuo*, leaving the white powdery product (343.4 mg, 0.59 mmol) in 88% yield. Crystals of (I) were grown by vapor diffusion of hexanes into THF.

### Refinement

The H-atoms were included in the refinements at geometrically idealized positions with C—H distances 0.98 and 0.99 Å for CH_3_ and CH_2_ type H-atoms, respectively; *U*_iso_ values were 1.5*U*_eq_ and 1.2*U*_eq_ of the carrier atoms for the methyl and CH_2_ groups, respectively. The final difference map showed a residual electron density in the vicinity of H31A atom and was chemically meaningless.

### Crystal data

**Table:**

[PtCl_2_(C_18_H_40_P_2_)]	*F*_000_ = 1160
*M**_r_* = 584.43	*D*_x_ = 1.663 Mg m^−^^3^
Monoclinic, *P*2_1_/*n*	Mo *K*α radiation λ = 0.71073 Å
Hall symbol: -P 2yn	Cell parameters from 4040 reflections
*a* = 11.0981 (10) Å	θ = 3.0–32.9º
*b* = 15.3242 (13) Å	µ = 6.38 mm^−^^1^
*c* = 14.5413 (13) Å	*T* = 100.0 (1) K
β = 109.287 (1)º	Block, colorless
*V* = 2334.2 (4) Å^3^	0.20 × 0.14 × 0.08 mm
*Z* = 4	

### Data collection

**Table:**

Bruker SMART APEXII CCD diffractometer	8022 independent reflections
Radiation source: fine-focus sealed tube	6312 reflections with *I* > 2σ(*I*)
Monochromator: graphite	*R*_int_ = 0.034
*T* = 100.0(1) K	θ_max_ = 32.0º
area detector, ω scans per φ	θ_min_ = 2.0º
Absorption correction: multi-scan(SADABS; Sheldrick, 2007)	*h* = −15→16
*T*_min_ = 0.342, *T*_max_ = 0.600	*k* = −22→22
20415 measured reflections	*l* = −19→21

### Refinement

**Table:**

Refinement on *F*^2^	Secondary atom site location: difference Fourier map
Least-squares matrix: full	Hydrogen site location: inferred from neighbouring sites
*R*[*F*^2^ > 2σ(*F*^2^)] = 0.029	H-atom parameters constrained
*wR*(*F*^2^) = 0.060	*w* = 1/[σ^2^(*F*_o_^2^) + (0.0232*P*)^2^] where *P* = (*F*_o_^2^ + 2*F*_c_^2^)/3
*S* = 1.02	(Δ/σ)_max_ = 0.002
8022 reflections	Δρ_max_ = 1.11 e Å^−^^3^
208 parameters	Δρ_min_ = −0.81 e Å^−^^3^
Primary atom site location: structure-invariant direct methods	Extinction correction: none

### Special details

**Table:**

Experimental. ^1^H NMR (CDCl_3_, 20 °C): δ 1.5 (d, ^3^*J*_HP_ = 14.1 Hz, 36 H, -(CH_3_)_3_), 1.9 (d, ^2^*J*_HP_ = 16 Hz, 4 H, -CH_2_-); ^13^C NMR (CDCl_3_, 20 °C): δ 24.5 (d, ^1^*J*_CP_ = 33 Hz, -CH_2_-), 30.4 (s, -(CH_3_)_3_), 37.6 (d, ^1^*J*_CP_ = 30 Hz, -C-); ^31^P NMR (CDCl_3_, 20 °C): δ 75.7 (s, with platinum satellites ^1^*J*_PPt_ = 3643.2 Hz).
Geometry. All e.s.d.'s (except the e.s.d. in the dihedral angle between two l.s. planes)are estimated using the full covariance matrix. The cell e.s.d.'s are takeninto account individually in the estimation of e.s.d.'s in distances, anglesand torsion angles; correlations between e.s.d.'s in cell parameters are onlyused when they are defined by crystal symmetry. An approximate (isotropic)treatment of cell e.s.d.'s is used for estimating e.s.d.'s involving l.s. planes.
Refinement. Refinement of *F*^2^ against ALL reflections. The weighted *R*-factor *wR* andgoodness of fit *S* are based on *F*^2^, conventional *R*-factors *R* are basedon *F*, with *F* set to zero for negative *F*^2^. The threshold expression of*F*^2^ > σ(*F*^2^) is used only for calculating *R*-factors(gt) *etc*. and isnot relevant to the choice of reflections for refinement. *R*-factors basedon *F*^2^ are statistically about twice as large as those based on *F*, and *R*-factors based on ALL data will be even larger.

### Fractional atomic coordinates and isotropic or equivalent isotropic displacement parameters (Å^2^)

**Table:**

	*x*	*y*	*z*	*U*_iso_*/*U*_eq_	
Pt1	0.268368 (10)	0.706474 (7)	0.781256 (8)	0.01505 (3)	
Cl1	0.17379 (7)	0.80168 (5)	0.86681 (6)	0.02422 (16)	
Cl2	0.44219 (8)	0.80401 (5)	0.81687 (6)	0.02415 (16)	
P1	0.09956 (7)	0.61552 (5)	0.74524 (5)	0.01603 (14)	
P2	0.36188 (7)	0.61746 (5)	0.70173 (6)	0.01776 (15)	
C1	0.0577 (3)	0.5718 (2)	0.8529 (2)	0.0227 (6)	
C2	−0.0053 (4)	0.4814 (2)	0.8323 (3)	0.0323 (8)	
H2A	−0.0253	0.4613	0.8896	0.048*	
H2B	0.0533	0.4400	0.8177	0.048*	
H2C	−0.0842	0.4851	0.7763	0.048*	
C3	−0.0326 (3)	0.6333 (2)	0.8823 (3)	0.0287 (7)	
H3A	−0.0520	0.6085	0.9380	0.043*	
H3B	−0.1120	0.6402	0.8273	0.043*	
H3C	0.0083	0.6903	0.9002	0.043*	
C4	0.1821 (3)	0.5634 (2)	0.9392 (2)	0.0308 (8)	
H4A	0.1632	0.5409	0.9960	0.046*	
H4B	0.2227	0.6208	0.9547	0.046*	
H4C	0.2400	0.5231	0.9221	0.046*	
C5	−0.0478 (3)	0.6578 (2)	0.6491 (2)	0.0232 (7)	
C6	−0.1592 (3)	0.5927 (2)	0.6247 (3)	0.0302 (8)	
H6A	−0.2334	0.6177	0.5744	0.045*	
H6B	−0.1812	0.5807	0.6834	0.045*	
H6C	−0.1342	0.5383	0.6006	0.045*	
C7	−0.0919 (3)	0.7459 (2)	0.6749 (3)	0.0316 (8)	
H7A	−0.1686	0.7645	0.6224	0.047*	
H7B	−0.0240	0.7891	0.6833	0.047*	
H7C	−0.1113	0.7406	0.7357	0.047*	
C8	−0.0127 (3)	0.6711 (3)	0.5569 (3)	0.0388 (9)	
H8A	−0.0871	0.6932	0.5046	0.058*	
H8B	0.0143	0.6154	0.5370	0.058*	
H8C	0.0573	0.7133	0.5700	0.058*	
C9	0.5007 (3)	0.5527 (2)	0.7849 (2)	0.0246 (7)	
C10	0.5670 (4)	0.4957 (2)	0.7285 (3)	0.0349 (9)	
H10A	0.6377	0.4637	0.7747	0.052*	
H10B	0.5999	0.5329	0.6874	0.052*	
H10C	0.5053	0.4541	0.6874	0.052*	
C11	0.5985 (3)	0.6126 (2)	0.8564 (3)	0.0340 (8)	
H11A	0.6690	0.5774	0.8984	0.051*	
H11B	0.5573	0.6440	0.8967	0.051*	
H11C	0.6317	0.6546	0.8199	0.051*	
C12	0.4469 (3)	0.4907 (2)	0.8451 (3)	0.0298 (8)	
H12A	0.5166	0.4561	0.8890	0.045*	
H12B	0.3840	0.4516	0.8011	0.045*	
H12C	0.4058	0.5249	0.8834	0.045*	
C13	0.4076 (3)	0.6704 (2)	0.5996 (2)	0.0241 (7)	
C14	0.5394 (3)	0.7143 (2)	0.6356 (3)	0.0292 (7)	
H14A	0.5579	0.7404	0.5802	0.044*	
H14B	0.6046	0.6707	0.6667	0.044*	
H14C	0.5396	0.7600	0.6829	0.044*	
C15	0.4083 (4)	0.6035 (2)	0.5199 (3)	0.0374 (9)	
H15A	0.4322	0.6329	0.4687	0.056*	
H15B	0.3230	0.5780	0.4919	0.056*	
H15C	0.4702	0.5572	0.5489	0.056*	
C16	0.3071 (3)	0.7402 (2)	0.5518 (3)	0.0286 (7)	
H16A	0.3286	0.7684	0.4988	0.043*	
H16B	0.3055	0.7839	0.6006	0.043*	
H16C	0.2229	0.7126	0.5256	0.043*	
C31	0.1465 (3)	0.5180 (2)	0.6924 (2)	0.0216 (6)	
H31A	0.1853	0.4755	0.7452	0.026*	
H31B	0.0690	0.4908	0.6465	0.026*	
C32	0.2411 (3)	0.5363 (2)	0.6385 (2)	0.0211 (6)	
H32A	0.1938	0.5577	0.5721	0.025*	
H32B	0.2842	0.4813	0.6320	0.025*	

### Atomic displacement parameters (Å^2^)

**Table:**

	*U*^11^	*U*^22^	*U*^33^	*U*^12^	*U*^13^	*U*^23^
Pt1	0.01739 (5)	0.01316 (5)	0.01369 (5)	0.00118 (5)	0.00389 (4)	−0.00008 (4)
Cl1	0.0232 (4)	0.0226 (4)	0.0254 (4)	0.0034 (3)	0.0061 (3)	−0.0079 (3)
Cl2	0.0275 (4)	0.0195 (4)	0.0271 (4)	−0.0052 (3)	0.0112 (3)	−0.0035 (3)
P1	0.0182 (4)	0.0177 (4)	0.0132 (3)	−0.0006 (3)	0.0066 (3)	−0.0002 (3)
P2	0.0202 (4)	0.0158 (4)	0.0199 (4)	−0.0004 (3)	0.0102 (3)	−0.0011 (3)
C1	0.0277 (16)	0.0264 (16)	0.0185 (15)	0.0043 (13)	0.0137 (13)	0.0043 (12)
C2	0.046 (2)	0.0275 (18)	0.0333 (19)	−0.0032 (16)	0.0261 (17)	0.0057 (15)
C3	0.0365 (19)	0.0297 (18)	0.0269 (17)	0.0024 (15)	0.0201 (15)	0.0022 (14)
C4	0.0348 (19)	0.041 (2)	0.0186 (16)	0.0045 (16)	0.0116 (14)	0.0099 (15)
C5	0.0185 (15)	0.0330 (18)	0.0165 (15)	−0.0029 (13)	0.0034 (12)	0.0040 (13)
C6	0.0208 (16)	0.037 (2)	0.0302 (19)	−0.0070 (14)	0.0053 (14)	−0.0058 (15)
C7	0.0222 (17)	0.0323 (19)	0.034 (2)	0.0028 (14)	0.0009 (15)	0.0085 (16)
C8	0.0208 (17)	0.071 (3)	0.0212 (18)	−0.0038 (17)	0.0018 (14)	0.0117 (18)
C9	0.0239 (16)	0.0186 (15)	0.0326 (18)	0.0045 (12)	0.0109 (14)	0.0015 (13)
C10	0.0326 (19)	0.0228 (17)	0.056 (2)	0.0042 (15)	0.0233 (18)	−0.0002 (16)
C11	0.0236 (17)	0.0309 (18)	0.041 (2)	0.0022 (15)	0.0021 (15)	0.0048 (16)
C12	0.0287 (18)	0.0263 (17)	0.036 (2)	0.0079 (14)	0.0126 (15)	0.0104 (15)
C13	0.0296 (17)	0.0250 (16)	0.0238 (17)	−0.0025 (14)	0.0172 (14)	−0.0011 (13)
C14	0.0272 (17)	0.0292 (18)	0.0363 (19)	0.0001 (14)	0.0173 (15)	0.0008 (15)
C15	0.050 (2)	0.042 (2)	0.0306 (19)	−0.0120 (19)	0.0282 (18)	−0.0071 (17)
C16	0.0315 (18)	0.0327 (18)	0.0255 (18)	−0.0035 (15)	0.0148 (15)	0.0084 (14)
C31	0.0270 (16)	0.0180 (15)	0.0243 (16)	−0.0022 (12)	0.0144 (13)	−0.0048 (12)
C32	0.0244 (15)	0.0202 (15)	0.0221 (15)	−0.0033 (12)	0.0125 (12)	−0.0046 (12)

### Geometric parameters (Å, °)

**Table:**

Pt1—P2	2.2513 (8)	C8—H8B	0.9800
Pt1—P1	2.2536 (8)	C8—H8C	0.9800
Pt1—Cl2	2.3588 (8)	C9—C11	1.535 (5)
Pt1—Cl1	2.3750 (8)	C9—C10	1.542 (5)
P1—C31	1.833 (3)	C9—C12	1.540 (5)
P1—C5	1.881 (3)	C10—H10A	0.9800
P1—C1	1.897 (3)	C10—H10B	0.9800
P2—C32	1.837 (3)	C10—H10C	0.9800
P2—C9	1.895 (3)	C11—H11A	0.9800
P2—C13	1.903 (3)	C11—H11B	0.9800
C1—C4	1.534 (5)	C11—H11C	0.9800
C1—C3	1.535 (4)	C12—H12A	0.9800
C1—C2	1.537 (5)	C12—H12B	0.9800
C2—H2A	0.9800	C12—H12C	0.9800
C2—H2B	0.9800	C13—C16	1.536 (5)
C2—H2C	0.9800	C13—C14	1.536 (5)
C3—H3A	0.9800	C13—C15	1.549 (5)
C3—H3B	0.9800	C14—H14A	0.9800
C3—H3C	0.9800	C14—H14B	0.9800
C4—H4A	0.9800	C14—H14C	0.9800
C4—H4B	0.9800	C15—H15A	0.9800
C4—H4C	0.9800	C15—H15B	0.9800
C5—C7	1.525 (5)	C15—H15C	0.9800
C5—C8	1.528 (5)	C16—H16A	0.9800
C5—C6	1.536 (4)	C16—H16B	0.9800
C6—H6A	0.9800	C16—H16C	0.9800
C6—H6B	0.9800	C31—C32	1.529 (4)
C6—H6C	0.9800	C31—H31A	0.9900
C7—H7A	0.9800	C31—H31B	0.9900
C7—H7B	0.9800	C32—H32A	0.9900
C7—H7C	0.9800	C32—H32B	0.9900
C8—H8A	0.9800		
			
P2—Pt1—P1	89.70 (3)	C5—C8—H8C	109.5
P2—Pt1—Cl2	90.82 (3)	H8A—C8—H8C	109.5
P1—Pt1—Cl2	178.77 (3)	H8B—C8—H8C	109.5
P2—Pt1—Cl1	178.84 (3)	C11—C9—C10	110.2 (3)
P1—Pt1—Cl1	91.39 (3)	C11—C9—C12	107.7 (3)
Cl2—Pt1—Cl1	88.09 (3)	C10—C9—C12	107.1 (3)
C31—P1—C5	105.46 (15)	C11—C9—P2	111.3 (2)
C31—P1—C1	103.85 (14)	C10—C9—P2	112.8 (2)
C5—P1—C1	110.23 (14)	C12—C9—P2	107.4 (2)
C31—P1—Pt1	105.77 (10)	C9—C10—H10A	109.5
C5—P1—Pt1	114.31 (11)	C9—C10—H10B	109.5
C1—P1—Pt1	115.98 (11)	H10A—C10—H10B	109.5
C32—P2—C9	105.70 (15)	C9—C10—H10C	109.5
C32—P2—C13	103.69 (14)	H10A—C10—H10C	109.5
C9—P2—C13	110.56 (15)	H10B—C10—H10C	109.5
C32—P2—Pt1	106.44 (10)	C9—C11—H11A	109.5
C9—P2—Pt1	113.88 (11)	C9—C11—H11B	109.5
C13—P2—Pt1	115.45 (11)	H11A—C11—H11B	109.5
C4—C1—C3	108.6 (3)	C9—C11—H11C	109.5
C4—C1—C2	108.2 (3)	H11A—C11—H11C	109.5
C3—C1—C2	108.1 (3)	H11B—C11—H11C	109.5
C4—C1—P1	107.8 (2)	C9—C12—H12A	109.5
C3—C1—P1	111.9 (2)	C9—C12—H12B	109.5
C2—C1—P1	112.1 (2)	H12A—C12—H12B	109.5
C1—C2—H2A	109.5	C9—C12—H12C	109.5
C1—C2—H2B	109.5	H12A—C12—H12C	109.5
H2A—C2—H2B	109.5	H12B—C12—H12C	109.5
C1—C2—H2C	109.5	C16—C13—C14	108.5 (3)
H2A—C2—H2C	109.5	C16—C13—C15	107.8 (3)
H2B—C2—H2C	109.5	C14—C13—C15	107.8 (3)
C1—C3—H3A	109.5	C16—C13—P2	107.9 (2)
C1—C3—H3B	109.5	C14—C13—P2	113.0 (2)
H3A—C3—H3B	109.5	C15—C13—P2	111.7 (2)
C1—C3—H3C	109.5	C13—C14—H14A	109.5
H3A—C3—H3C	109.5	C13—C14—H14B	109.5
H3B—C3—H3C	109.5	H14A—C14—H14B	109.5
C1—C4—H4A	109.5	C13—C14—H14C	109.5
C1—C4—H4B	109.5	H14A—C14—H14C	109.5
H4A—C4—H4B	109.5	H14B—C14—H14C	109.5
C1—C4—H4C	109.5	C13—C15—H15A	109.5
H4A—C4—H4C	109.5	C13—C15—H15B	109.5
H4B—C4—H4C	109.5	H15A—C15—H15B	109.5
C7—C5—C8	107.0 (3)	C13—C15—H15C	109.5
C7—C5—C6	109.4 (3)	H15A—C15—H15C	109.5
C8—C5—C6	107.7 (3)	H15B—C15—H15C	109.5
C7—C5—P1	113.1 (2)	C13—C16—H16A	109.5
C8—C5—P1	106.7 (2)	C13—C16—H16B	109.5
C6—C5—P1	112.6 (2)	H16A—C16—H16B	109.5
C5—C6—H6A	109.5	C13—C16—H16C	109.5
C5—C6—H6B	109.5	H16A—C16—H16C	109.5
H6A—C6—H6B	109.5	H16B—C16—H16C	109.5
C5—C6—H6C	109.5	C32—C31—P1	113.8 (2)
H6A—C6—H6C	109.5	C32—C31—H31A	108.8
H6B—C6—H6C	109.5	P1—C31—H31A	108.8
C5—C7—H7A	109.5	C32—C31—H31B	108.8
C5—C7—H7B	109.5	P1—C31—H31B	108.8
H7A—C7—H7B	109.5	H31A—C31—H31B	107.7
C5—C7—H7C	109.5	C31—C32—P2	112.3 (2)
H7A—C7—H7C	109.5	C31—C32—H32A	109.2
H7B—C7—H7C	109.5	P2—C32—H32A	109.2
C5—C8—H8A	109.5	C31—C32—H32B	109.2
C5—C8—H8B	109.5	P2—C32—H32B	109.2
H8A—C8—H8B	109.5	H32A—C32—H32B	107.9
			
P2—Pt1—P1—C31	8.74 (11)	C1—P1—C5—C6	−48.9 (3)
Cl1—Pt1—P1—C31	−170.88 (11)	Pt1—P1—C5—C6	178.4 (2)
P2—Pt1—P1—C5	−106.82 (12)	C32—P2—C9—C11	−169.3 (2)
Cl1—Pt1—P1—C5	73.55 (12)	C13—P2—C9—C11	79.1 (3)
P2—Pt1—P1—C1	123.22 (11)	Pt1—P2—C9—C11	−52.8 (3)
Cl1—Pt1—P1—C1	−56.40 (11)	C32—P2—C9—C10	66.3 (3)
P1—Pt1—P2—C32	9.20 (11)	C13—P2—C9—C10	−45.3 (3)
Cl2—Pt1—P2—C32	−169.68 (11)	Pt1—P2—C9—C10	−177.3 (2)
P1—Pt1—P2—C9	−106.85 (12)	C32—P2—C9—C12	−51.6 (2)
Cl2—Pt1—P2—C9	74.27 (12)	C13—P2—C9—C12	−163.2 (2)
P1—Pt1—P2—C13	123.65 (12)	Pt1—P2—C9—C12	64.9 (2)
Cl2—Pt1—P2—C13	−55.24 (12)	C32—P2—C13—C16	81.9 (2)
C31—P1—C1—C4	83.6 (2)	C9—P2—C13—C16	−165.2 (2)
C5—P1—C1—C4	−163.8 (2)	Pt1—P2—C13—C16	−34.1 (3)
Pt1—P1—C1—C4	−31.9 (3)	C32—P2—C13—C14	−158.1 (2)
C31—P1—C1—C3	−157.1 (2)	C9—P2—C13—C14	−45.2 (3)
C5—P1—C1—C3	−44.5 (3)	Pt1—P2—C13—C14	85.9 (2)
Pt1—P1—C1—C3	87.4 (2)	C32—P2—C13—C15	−36.4 (3)
C31—P1—C1—C2	−35.4 (3)	C9—P2—C13—C15	76.5 (3)
C5—P1—C1—C2	77.2 (3)	Pt1—P2—C13—C15	−152.4 (2)
Pt1—P1—C1—C2	−151.0 (2)	C5—P1—C31—C32	91.5 (2)
C31—P1—C5—C7	−172.7 (2)	C1—P1—C31—C32	−152.5 (2)
C1—P1—C5—C7	75.7 (3)	Pt1—P1—C31—C32	−30.0 (2)
Pt1—P1—C5—C7	−57.0 (3)	P1—C31—C32—P2	39.6 (3)
C31—P1—C5—C8	−55.3 (3)	C9—P2—C32—C31	91.6 (2)
C1—P1—C5—C8	−166.8 (2)	C13—P2—C32—C31	−152.0 (2)
Pt1—P1—C5—C8	60.4 (3)	Pt1—P2—C32—C31	−29.8 (2)
C31—P1—C5—C6	62.6 (3)		
